# Comparative Genomics and Transcriptomics During Sexual Development Gives Insight Into the Life History of the Cosmopolitan Fungus *Fusarium neocosmosporiellum*

**DOI:** 10.3389/fmicb.2019.01247

**Published:** 2019-06-07

**Authors:** Wonyong Kim, Brad Cavinder, Robert H. Proctor, Kerry O’Donnell, Jeffrey P. Townsend, Frances Trail

**Affiliations:** ^1^Department of Plant Biology, Michigan State University, East Lansing, MI, United States; ^2^Mycotoxin Prevention and Applied Microbiology Research Unit, United States Department of Agriculture, Peoria, IL, United States; ^3^Department of Biostatistics, Yale University, New Haven, CT, United States; ^4^Department of Ecology and Evolutionary Biology, Yale University, New Haven, CT, United States; ^5^Department of Plant, Soil and Microbial Sciences, Michigan State University, East Lansing, MI, United States

**Keywords:** perithecium, *Fusarium*, transcriptome, mating type locus, secondary metabolic genes, sexual development

## Abstract

*Fusarium neocosmosporiellum* (formerly *Neocosmospora vasinfecta*) is a cosmopolitan fungus that has been reported from soil, herbivore dung, and as a fruit- and root-rot pathogen of numerous field crops, although it is not known to cause significant losses on any crop. Taking advantage of the fact that this species produces prolific numbers of perithecia in culture, the genome of *F. neocosmosporiellum* was sequenced and transcriptomic analysis across five stages of perithecium development was performed to better understand the metabolic potential for sexual development and gain insight into its life history. Perithecium morphology together with the genome and transcriptome were compared with those of the plant pathogen *F. graminearum*, a model for studying perithecium development. Larger ascospores of *F. neocosmosporiellum* and their tendency to discharge as a cluster demonstrated a duality of dispersal: the majority are passively dispersed through the formation of cirrhi, while a minority of spores are shot longer distances than those of *F. graminearum.* The predicted gene number in the *F. neocosmosporiellum* genome was similar to that in *F. graminearum*, but *F. neocosmosporiellum* had more carbohydrate metabolism-related and transmembrane transport genes. Many transporter genes were differentially expressed during perithecium development in *F. neocosmosporiellum*, which may account for its larger perithecia. Comparative analysis of the secondary metabolite gene clusters identified several polyketide synthase genes that were induced during later stages of perithecium development. Deletion of a polyketide synthase gene in *F. neocosmosporiellum* resulted in a defective perithecium phenotype, suggesting an important role of the corresponding metabolite, which has yet to be identified, in perithecium development. Results of this study have provided novel insights into the genomic underpinning of development in *F. neocosmosporiellum*, which may help elucidate its ability to occupy diverse ecological niches.

## Introduction

Flask-shaped fruiting bodies called perithecia predominate in the class Sordariomycetes within the Ascomycota of the Kingdom Fungi. This class contains some of the most problematic plant and insect pathogens, and saprotrophs, including the genetic model *Neurospora crassa*. Evolution has bequeathed evolvable features to the perithecia of Sordariomycetes, conveying the ability to disperse spores most efficiently according to niche (Trail, 2007). The genomics of perithecium production has been most extensively studied in *Fusarium graminearum* and *N. crassa*. *F. graminearum* is an important pathogen of grain crops and perithecia contribute significantly to its disease cycle (Desjardins et al., 2006). Genes involved in perithecium development have been identified through transcriptomics and genomics studies, leading to large-scale gene function analyses (Son et al., 2011; Wang et al., 2012; Trail and Gardiner, 2014). Most recently, a study of the ancestral levels of transcription in *Fusarium* and *Neurospora* species have revealed genes that play significant roles in their divergent morphologies (Trail et al., 2017).

Ascospores are the products of meiosis and are formed within perithecia in the majority of Sordariomycetes. Typically eight ascospores are formed within an ascus, which when fully developed provides sufficient turgor pressure to forcibly discharge ascospores into the air above perithecia, thereby dispersing them. Ascospores vary in size, shape, ornamentation, and pigmentation. These features can affect dispersal distance, survival in air due to UV damage, and other factors (Trail, 2007). Species are homothallic (self-fertile) or heterothallic (self-sterile), and mating type is determined by the presence of *MAT1-1* and/or *MAT1-2* idiomorphs. Idiomorph is the term used to describe the two forms of the mating type locus in the Ascomycota. Within a species, *MAT1-1* and *MAT2-1* idiomorphs occur at the same genomic location, but they are not considered alleles, because genes in the two idiomorph types are not homologous (Metzenberg and Glass, 1990). Thus, in heterothallic *Fusarium* species, the *MAT1-1* idiomorph includes the *MAT1-1-1*, *MAT1-1-2*, and *MAT1-1-3* genes, while the *MAT1-2* idiomorph includes the *MAT1-2-1* and *MAT1-2-3* genes. In the homothallic species *F. graminearum*, by contrast, the *MAT* locus includes all five of these genes.

*Fusarium* species occupy a wide variety of niches, and include many important plant pathogens and a number of opportunistic human pathogens (O’Donnell et al., 2010), soil saprotrophs, insect parasites, and plant endophytes (Rodriguez et al., 2009). Fusaria are notorious for producing toxic secondary metabolites (mycotoxins) that can contaminate food and feed, rendering it unsafe for consumption by humans and other animals. Synthesis of these and other secondary metabolites typically involves non-ribosomal peptide synthase (NRPS), polyketide synthase (PKS), prenyltransferase, and/or terpene synthase enzymes that catalyze synthesis of structures that typically undergo one or more subsequent modifications. In fungi, genes encoding these enzymes are often found clustered with genes encoding modifying enzymes such as acetyltransferases, methyltransferases, dehydrogenases, and monooxygenases that are required for biosynthesis of the final products. Production of secondary metabolites is often associated with niche-specific adaptations (Turgeon and Bushley, 2010).

The *Fusarium sambucinum* (FSAMSC) and *F*. *solani* (FSSC) species complexes are among the most agriculturally important and species-rich lineages within the genus (O’Donnell et al., 2013). In prior research, we studied the homothallic, cereal pathogen *F. graminearum* (FSAMSC) with particular emphasis on development and function of perithecia, as well as production of mycotoxins and other secondary metabolites (Gaffoor et al., 2005; Sikhakolli et al., 2012; Wang et al., 2012, 2018; Trail et al., 2017; Kim et al., 2018). Recently, we began a study of perithecium development in homothallic *F. neocosmosporiellum* (formerly *Neocosmospora vasinfecta*; Geiser et al., 2013), a member of the FSSC that is reported to be pathogenic on peanut and soybean (Dau et al., 2010; Pan et al., 2010; Sun et al., 2014; Greer et al., 2015), and an opportunistic pathogen of humans (Cornely et al., 2001; Gabriel et al., 2013). This cosmopolitan species has also been reported on infected soybean cyst nematodes (Gintis et al., 1983) and dung of diverse herbivores (Calaça et al., 2013). It also possesses a highly active CO_2_ fixation mechanism (Budd, 1969). To better understand the metabolic potential of this fungus, we sequenced the genome of *F. neocosmosporiellum* NRRL 22166 and compared its gene content with previously published *F. graminearum* genomes. Both are homothallic and prolific producers of perithecia, but their niches, and perithecium and ascospore morphology are quite divergent. We performed transcriptomics across stages of perithecium development to better understand differences in morphology and function of the perithecia in these species.

## Materials and Methods

### Fungal Strain and Genome Sequencing

*Fusarium neocosmosporiellum* NRRL 22166 was isolated from the soybean cyst nematode *Heterodera glycines* in southern Illinois in 1983 (O’Donnell, 2000). To prepare DNA for genome sequencing, *F. neocosmosporiellum* was grown in YEPD medium (0.1% yeast extract, 0.1% peptone, 2% glucose) for 2 days at room temperature on a rotary shaker set at 200 rpm. Mycelia were harvested by filtration, lyophilized, ground to a powder, and genomic DNA was extracted using the ZR Fungal/Bacterial DNA MiniPrep kit (Zymo Research, Irvine, CA, United States). A DNA library was prepared from genomic DNA with the Nextera XT DNA library Preparation Kit using the protocol specified by the manufacturer (Illumina, Inc., San Diego, CA, United States). Sequence reads were generated from the library using a paired-end approach with a MiSeq Illumina platform (Illumina, Inc.). Reads were processed and assembled using CLC Genomics Workbench (Qiagen Inc.) (Supplementary Table S1). Completeness of the assembled genome was evaluated by BUSCO (v1.22) using ‘Sordariomyceta odb9’ database (Simão et al., 2015).

### Sexual Stage Induction and RNA-seq

*Fusarium neocosmosporiellum* was maintained on potato dextrose agar (PDA) plates, then grown in carboxymethylcellulose medium for 4 days to produce microconidia (Cappellini and Peterson, 1965). Carrot agar plates (60 mm in diameter) (Klittich and Leslie, 1988) were inoculated by spreading 50 μl of a 10^5^ spores/ml conidial suspension across the surface of the agar, which was incubated at room temperature under constant light. Two days after incubation, aerial hyphae and conidia were removed by gently scraping the culture surface with a spatula, and then 0.9 ml of 2.5% Tween 60 (Sigma-Aldrich, St. Louis, MO, United States) was applied to the surface to induce perithecia. Hyphae and perithecial tissues were collected when the six successive developmental stages delineated by Sikhakolli et al. (2012) were observed. The stages are as follows: S0–2 h after the Tween 60 application, which represents the immediate reaction to possible physical damage of hyphae and Tween 60, but does not reflect significant development; S1–formation of ascogonia (fertile hyphae that function as the ‘female’ during the sexual cycle, which are subsequently enveloped by perithecial tissues); S2–formation of perithecial walls; S3–formation of paraphyses (sterile hyphae interspersed with asci in a perithecium); S4–formation of asci (sac-like structures in which meiosis occurs followed by a synchronous round of mitosis); S5–formation of eight uniseriate ascospores.

Total RNA was extracted from hyphae and perithecial tissues ground in liquid nitrogen using TRIzol reagent (Thermo Fisher Scientific, Waltham, MA, United States) according to the manufacturer’s instructions together with the following extraction steps: two phenol (pH 4.6)-chloroform-isoamyl alcohol (25:24:1) extraction steps followed by two chloroform extraction steps after the initial TRIzol-chloroform phase separation. RNA pellets were dissolved in 88 μL of nuclease-free water and subjected to genomic DNA digestion with DNase (Qiagen Inc.). RNA samples were then concentrated using RNA Clean & Concentrator (Zymo Research). RNA quality was confirmed using an Agilent 2100 Bioanalyzer (Agilent Technologies, Palo Alto, CA, United States). Two micrograms of total RNA per sample was used for cDNA library construction. Strand-specific cDNA libraries were constructed from poly-A captured RNAs using the KAPA Stranded RNA-Seq Library Preparation Kit (Kapa Biosystems, Wilmington, MA, United States) and sequenced on an Illumina HiSeq 2500 platform (Illumina Inc.; single-end 50 bp reads) at Michigan State University’s Research Technology Support Facility^1^.

### Ascospore Release

Forcible spore discharge was studied as previously described (Trail et al., 2005). In brief, the horizontal distance that spores traveled was measured in a glass chamber to minimize free convection current (Aylor and Anagnostakis, 1991). Perithecium formation was induced on carrot agar so that the spores were ejected lengthwise down the coverslip, which covered the length of the 6 cm chamber. The discharged spores were examined by removing the coverslip on which the agar rested and the cover slip in the line of fire at the end of the chamber.

### RNA-seq Data Processing and Genome Annotation

The quality of raw reads was assessed with FastQC (v0.11.3)^2^. Poor quality reads were trimmed or filtered out, and adapters and homopolymers were trimmed from raw reads using ngsShoRT (v2.2; Chen et al., 2014) with option arguments: ‘-lqs 12’, ‘-tera_avg 20’, ‘-5a_mp 98’, and ‘-rmHP_ml 10.’ Filtered reads were mapped to the *F. neocosmosporiellum* genome using HISAT2 (v2.0.4; Kim D. et al., 2015). Functional annotation of the genome was performed using MAKER2 (Holt and Yandell, 2011). RNA-seq data for the six developmental stages was provided as transcript evidence to improve gene models prediction by MAKER2, yielding a total of 14,353 gene models. Ensembl annotation version 32 of the *F. graminearum* PH-1 genome was used, (accessions: FGSC9075/NRRL 31084) (King et al., 2015) as well as the transcriptome data during perithecium development (Kim et al., 2018). Orthologous genes shared by the two fungi were grouped by OrthoMCL using an inflation value of 2.5 (Li et al., 2003).

### Identification of CAZyme Genes and Secondary Metabolite Biosynthetic Gene Clusters

Carbohydrate-active enzyme (CAZyme) families involved in the breakdown, biosynthesis, or modification of carbohydrates and glycoconjugates (Lombard et al., 2014) were detected by running *hmmscan* in HMMER (v3.1b2; Eddy, 2011), with an *E*-value cutoff of 1 × 10^-10^. Hidden Markov model (HMM) profiles for CAZyme families were acquired from the dbCAN v6.0 database (Huang et al., 2018). When overlapping modules were detected, the one with the lowest *E*-value was retained. Genes annotated as one of the four CAZyme families (GH, GT, PL, and CE) were included, and the predicted CAZyme genes with a covered fraction of HMMs lower than 0.30 were excluded. Secondary metabolite biosynthetic gene clusters were predicted using antiSMASH (v4.0.2; Medema et al., 2011) with a default setting (accessed November 2, 2017). Of the 35 biosynthetic gene clusters identified in the *F. neocosmosporiellum* genome, we analyzed the type I-PKS, NRPS, and terpene gene clusters.

### Differential Expression and Functional Enrichment Analyses

Read counts for the annotated genes were calculated using *htseq-count* (v0.6.1; Anders et al., 2015) in *F. graminearum* and *F. neocosmosporiellum*. Gene expression levels in counts-per-million (CPM) values were computed and normalized by effective library size estimated by ‘trimmed mean of *M*-values’ using edgeR (v3.14.0; Robinson et al., 2010). We defined expressed genes when a gene had CPM values greater than 1 in at least 3 samples. Expressed genes with log_2_[fold-change] greater than 1.5 between two successive developmental stages were identified as differentially expressed (DE) at a 5% false discovery rate (FDR) using limma (v3.28.21; Law et al., 2016). Gene ontology (GO) terms were assigned to the genomes of the two fungi using Trinotate (v3.0.1; Bryant et al., 2017). The list of GO terms was customized by adding several GO terms related to developmental processes to the GO Slim terms specific for fission yeast as described in Kim et al. (2018). Functional enrichment analyses for DE genes were performed using GOseq (v1.24.0), including only those genes annotated by one or more GO terms (Young et al., 2010). To assess enrichment of GO terms, the Wallenius approximation, which is an extension of the hypergeometric distribution, and the Benjamini–Hochberg method were used to calculate the FDR-corrected (adjusted) *P*-value.

### Coexpression Network Analysis

The weighted gene correlation network analysis (WGCNA) R package (v1.51; Langfelder and Horvath, 2008) was used to cluster CAZyme genes by averaged RPKM values across developmental stages. The ‘*pickSoftThreshold*’ function was used to determine soft-thresholding power that measures the strength of correlation based on the direct correlation value of pairs of genes and the weighted correlations of all of their shared neighbors in the network space. The soft-thresholding power 28 and 15 were selected, respectively, for *F. graminearum* and *F. neocosmosporiellum* CAZyme genes. A range of treecut values was tested for cluster detection, and the value was set to 0.18, which corresponding to a correlation of 0.82. All other WGCNA parameters were set at the default.

### Generation of Targeted Gene-Deletion Mutants

To generate gene replacement mutants, we used double-joint PCR and split-marker strategies with a minor modification (Catlett et al., 2003; Yu et al., 2004). For the first round of PCR, upstream (left flanking) regions and downstream (right flanking) regions of the coding sequence of target genes (*PKS7*, *PKS35* and *TC2*) were amplified using L5 and L3 primer pairs and R5 and R3 primer pairs, respectively. L3 and R5 primers have 27 nt-long overhang sequences complementary to the 5′ and 3′ ends of a 1,376-bp hygromycin phosphotransferase gene (*hph*) cassette that was amplified from the pCB1004 plasmid (Carroll et al., 1994) using the HYG5 and HYG3 primers. In the second round of PCR, left and right flanking regions were fused to the *hph* cassette through PCR by overlap extension. For amplification of split marker constructs, one microliter of the PCR product from the second round was used as template for the third round of PCR with the following nested primer pairs: N5 and HY-R pairs to generate 5′-split constructs, and YG-F and N3 pairs to generate 3′-split constructs. The split marker constructs were introduced into protoplasts by polyethylene glycol-mediated genetic transformation as described in Hallen-Adams et al. (2011). Target gene replacement with the *hph* cassette was verified in transformants by diagnostic PCRs (Supplementary Figure S1).

Genetic complementation of the Δ*PKS7* strain was accomplished by introducing the *PKS7* locus, which includes the *PKS7* coding sequence plus 700 and 300 bp of the upstream and downstream sequences, respectively. The 5′ half (3,498 bp) and 3′ half (6,287 bp) of the *PKS7* gene locus were amplified separately using primer sets PKS7-5H and PKS7-3H, respectively. Then, the two PCR fragments and the pDS23 plasmid (Teichert et al., 2012), which were precut with *Avr*II and *Bgl*II, were assembled using a Gibson Assembly reagent (New England Biolabs Inc., Ipswich, MA, United States). Importantly, the two fragments of the *PKS7* gene were designed to be assembled at an intron region to avoid a possible frameshift due to misassembly. Reintroduction of the *PKS7* gene locus was confirmed by PCR (Supplementary Figure S1). Primers used in targeted gene knockouts and genetic complementation are listed in Supplementary Table S2.

### Data Accession

The *F. neocosmosporiellum* NRRL 22166 genome sequence can be accessed through accession number SSHR0000000 in NCBI. The RNA-seq data generated in the present study was deposited in NCBI’s Gene Expression Omnibus and is accessible through GEO series accession number GSE124553.

## Results

### Perithecium Development, Genome, and Transcriptomes of *F. neocosmosporiellum*

**Figure 1:**
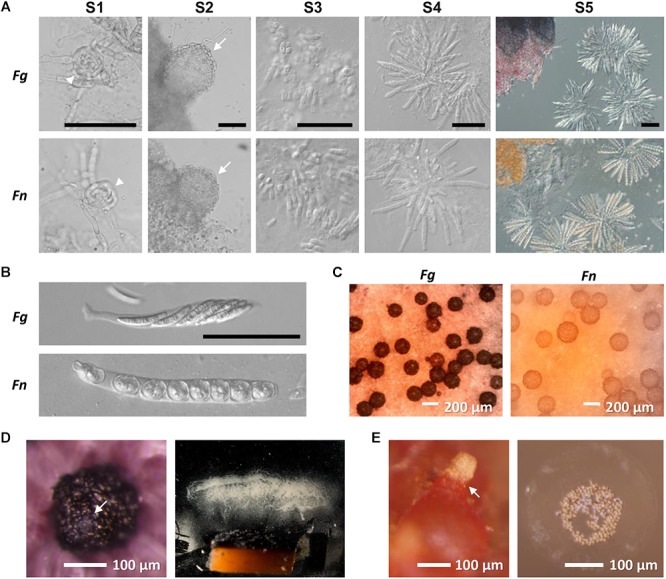
Perithecium development in *F. graminearum* (*Fg*) and *F. neocosmosporiellum* (*Fn*). **(A)** sexual stage definition. S1—ascogonium (arrowheads), S2—protoperithecia with conspicuous perithecial wall layers (arrows), S3—paraphyses (knuckle-like cells) released from squashed perithecium, S4—asci released from squashed perithecium, and S5—asci filled with ascospores. Scaling is the same for each stage in both species (Bar = 50 μm). **(B)** Ascus and ascospore morphology. **(C)** Mature perithecia grown on carrot agar. **(D)** Perithecium of *Fg* with cirrhus oozing from ostiole (arrow; left panel) and spore print from agar block containing numerous perithecia (right panel). **(E)** Perithecium of *Fn* with cirrhus oozing from ostiole (arrow; left panel) and spores fired in groups with visible halo of mucilage (right panel).

*De novo* sequence assembly of MiSeq-generated reads of *F. neocosmosporiellum* resulted in 50× coverage and an estimated genome size of 54,657,069 bp. The GC content of the *F. neocosmosporiellum* genome (51.4%) was slightly higher than that of *F. graminearum* (48.3%), but it is comparable to a closely-related species reported as *F. solani* f. sp. *pisi* (51.7%) (Coleman et al., 2009). Similar to other *Fusarium* species (King et al., 2018), a total of 14,353 genes were predicted using the MAKER2 gene modeling program (Holt and Yandell, 2011). Completeness of the sequenced genome was quantified by the presence of core eukaryotic genes and was shown to be comparable to that of the finished *F. graminearum* genome and other related species (Table 1 and Supplementary Figure S2). To compare the transcriptional profile of *F. neocosmosporiellum* to that of *F. graminearum* during perithecium development, *F. neocosmosporiellum* RNA samples from vegetative hyphae after sexual induction (S0) and from five successive sexual stages (S1–S5) were sequenced and used in the comparative studies (Figure 1A). Mature perithecia of both species containing asci with ascospores are compared in Figure 1B,C. A total of 680 million RNA-seq reads were generated from 18 samples (6 stages × 3 replicates), with an average of 37 million mapped reads per sample (Supplementary Figure S3).

**Table 1:** Genome summary and completeness.

	*F. graminearum^a^*	*F. neocosmosporiellum*
Genome size (bp)	36,563,796	54,657,069
GC (%)	48.0	51.4
Predicted genes (#)	14,164	14,353
Expressed genes (#)*^b^*	12,024	12,486
BUSCO complete (%)	96	93
BUSCO fragmented (%)	3	5
BUSCO missing (%)	1	2

^a^*Based on genome assembly version RRes v4.0 (King et al., 2015).*^b^*Filtered by an expression threshold (see Materials and Methods).*

### Ascospore Dispersal

Spore dispersal from mature perithecia took two forms. Spores forcibly discharged were primarily deposited on the far end of the chamber, with a few ascospores landing at the coverslip base and a few on the ceiling of the chamber. Compared to the quantity of discharged ascospores from *F. graminearum* (Figure 1D; Trail et al., 2005), the ascospore numbers that accumulated on the coverslip supporting perithecia were exceedingly low, amounting to less than 8 ascospores per perithecium. The vast majority of perithecia eventually exuded ascospores *en masse* in the form of cirrhi (Figure 1E). In addition, ascospores on the cover slip at the far end of the chamber indicated that spores landed in groups, suggesting that they likely remained together when ejected and during flight. A mucilaginous halo was visible surrounding groups of ascospores (Figure 1E). Attempts to dislodge the ascospores by adding a drop of water and leaving it up to 3 h were unsuccessful for the majority of the spores, as less than 10% dislodged.

### *MAT* Locus in *F. neocosmosporiellum*

**Figure 2:**
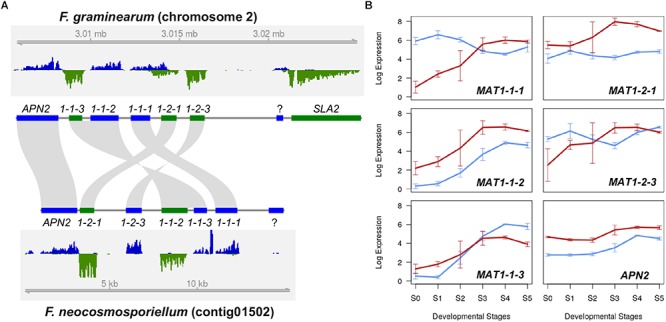
Mating type locus comparison **(A)** Structural organization of the *MAT* locus in *F. graminearum* and *F. neocosmosporiellum*. Genes that occur in both the *MAT1-1* and *MAT1-2* idiomorphs in heterothallic species occur together at a single *MAT* locus in these two homothallic species. In most ascomycetes, the *MAT* locus is flanked by *APN2* (encoding a DNA lyase) and *SLA2* (encoding a cytoskeletal protein), which are convergently transcribed toward the *MAT* genes. RNA-seq read coverage for *MAT* and flanking genes at S4 (ascus development stage) is plotted for both DNA strands (blue and green) with genome coordinates. Direction of transcription is the same for genes depicted as blue bars and opposite that of genes depicted as green bars. **(B)** *MAT* gene expression during perithecium development. Expression levels are presented as log_2_[RPKM+1]; Blue lines: *F. graminearum*, Red lines: *F. neocosmosporiellum*.

*Fusarium neocosmosporiellum* is homothallic (self-fertile), and sequence analysis indicated that its mating type locus includes both *MAT1-1* and *MAT1-2* genes. Although the *MAT* locus in *F. neocosmosporiellum* and *F. graminearum* include both *MAT1-1* and *MAT1-2* genes, the order of the *MAT* genes and their direction of transcription differs in these two homothallic species (Figure 2A). Organization of the *MAT* genes suggests that they have been rearranged by one or two inversions relative to closely related species in the FSSC. One inversion involved *MAT1-1-3* and *MAT1-1-2*, the order of which is inverted compared to closely related fusaria (Hughes et al., 2014), and the other inversion involved *MAT1-2-3*, which was divergently transcribed from *MAT1-2-1* in *F. neocosmosporiellum*, but not in closely related fusaria (Hughes et al., 2014). However, the ancestral state of the transcriptional direction of *MAT1-2* genes in the FSSC remains to be determined (Hughes et al., 2014).

In addition to differences in gene organization, expression of *MAT* genes during perithecium development differed in the two fungi. In *F. neocosmosporiellum*, expression of all five *MAT* genes appeared to be synchronized and gradually increased throughout perithecium development (Figure 2B). By contrast, the *MAT1-1-1*, *MAT1-2-1*, and *MAT1-2-3* in *F. graminearum* were already expressed at earlier stages, slightly decreased, then increased again at later stages (Figure 2B), which is in consistent with the previous expression studies, and real-time PCR data (Kim et al., 2012). Interestingly, our strand-specific RNA-seq data revealed that the *MAT1-1-1* and *MAT1-2-1* genes in *F. graminearum* possessed an extended 3′ untranslated region, and the convergently transcribed genes overlapped each other, while no such overlap was observed in *F. neocosmosporiellum* (Figure 2A).

### CAZyme Gene Expression During Perithecium Development

**Figure 3:**
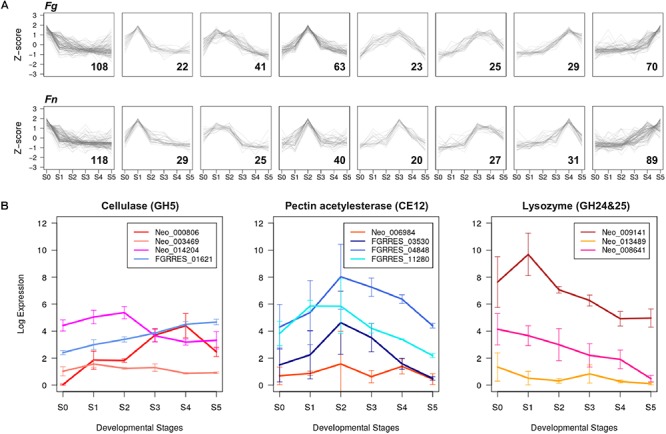
Developmental stage-specific expression of CAZyme genes **(A)** Co-expressed groups of CAZyme genes in *F. graminearum* (*Fg*) and *F. neocosmosporiellum* (*Fn*) represented in trend plots of *Z*-score normalized RPKM values (*y*-axis) in a given group across perithecium development (*x*-axis). Gene numbers in parentheses. **(B)** Expression profiles of GH5, CE12, GH24, and GH25 CAZyme families identified in *F. graminearum* and *F. neocosmosporiellum.* Expression levels are presented as log_2_[RPKM+1].

The annotated *F. neocosmosporiellum* genome was predicted to contain more genes classified as CAZymes (i.e., genes involved in the breakdown and modification of polysaccharides; Lombard et al., 2014), compared to *F. graminearum* (*E*-value < 10^-10^; Table 2). However, the perithecium transcriptome data indicated that the number of CAZyme genes expressed during perithecium development was similar in the two species (Table 2). Co-expression analyses showed that many of the expressed CAZyme genes were induced in different developmental stages in the two fusaria, suggesting they play unique roles during perithecium development (Figure 3A). The CAZyme genes in the two species comprised about 3.4% of the gene content. Although the percentage of CAZyme genes was similar in both, some differences between the species were observed.

**Table 2:** Annotation of expressed CAZyme in *Fusarium graminearum* (*Fg*) and *F. neocosmosporiellum* (*Fn*).

Classification*^a^*	CAZyme families (enzyme activity)	*Fg*	*Fn*
CAZymes (expressed)*^b^*	CE: Carbohydrate esterases	93	88
	GH: Glycoside hydrolases	224	211
	GT: Glycosyl transferases	81	91
	PL: Polysaccharide lyases	18	23
	Total	416 (481)*^c^*	413 (551)*^c^*
Cellulose	GH1 (β-glucosidase, etc.)	3	3
	GH5 (cellulase, etc.)	1	3
	GH6 (endoglucanase and cellobiohydrolase)	1	0
	GH45 (endoglucanase)	1	1
	GH74 (reducing end cellobiohydrolase)	1	1
	Subtotal	7	8
Pectin	CE8 (pectin methylesterase)	3	3
	CE12 (pectin acetylesterase)	3	1
	GH105 (rhamnogalacturonyl hydrolase, etc.)	3	2
	PL1 (pectate lyase, etc.)	8	9
	PL3 (pectate lyase)	6	7
	PL9 (hyaluronate lyase, etc.)	0	1
	Subtotal	23	23
Xylan and	CE1 (acetyl xylan esterase, etc.)	6	7
hemicellulose	CE2 (acetyl xylan esterase)	1	0
sidechain	CE3 (acetyl xylan esterase)	5	5
modifications	CE4 (acetyl xylan esterase, etc.)	6	6
	CE5 (acetyl xylan esterase and cutinase)	9	3
	GH10 (endo-β-1,4-xylanase, etc.)	1	1
	GH11 (endo-β-1,3-xylanase, etc.)	2	1
	GH43 (β-xylosidase, etc.)	12	12
	GH57 (α-amylase, etc.)	1	1
	GH62 (α-L-arabinofuranosidase)	1	0
	GH67 (α-glucuronidase, etc.)	1	0
	GH95 (α-L-fucosidase, etc.)	2	1
	GH115 (xylan α-1,2-glucuronidase etc.)	2	1
	Subtotal	49	38

^a^*CAZyme classification by targeting plant cell wall carbohydrate, as in Gruninger et al. (2018).*^b^*Filtered by an expression threshold (see Materials and Methods). *^*c*^*Total predicted CAZyme genes in parentheses (*E*-value < 10^*-10*^).*

*Fusarium neocosmosporiellum* possesses three genes in the CAZyme family GH5, which targets cellulose, while *F. graminearum* contained only one gene in this family (Table 2). Although one of the three GH5-family genes, Neo_003469, exhibited low expression levels throughout perithecium development, the other two genes, Neo_014204 and Neo_000806, were induced at early and late stages, respectively, of perithecium development (Figure 3B). Expression of the sole GH5-family gene in *F. graminearum* was relatively constitutive. *Fusarium graminearum* possesses three genes in the CAZyme family CE12, which targets pectin, while *F. neocosmosporiellum* contained only one gene in this family (Table 2). The CE12-family genes, FGRRES_03530, FGRRES_04848 and FGRRES_11280, in *F. graminearum* were highly induced at earlier developmental stages than the single CE12-family gene, Neo_006984, in *F. neocosmosporiellum* (Figure 3B). Also, *F. neocosmosporiellum* possesses three genes in the CAZyme families GH24 and GH25, which putatively target bacterial cell walls (i.e., peptidoglycan) (Zhao et al., 2013). These genes were expressed during earlier developmental stages, followed by gradual decline in expression during later stages (Figure 3B). The GH24- and GH25-family genes were absent in *F. graminearum.*

### Transcriptome Changes During Perithecium Development

**Figure 4:**
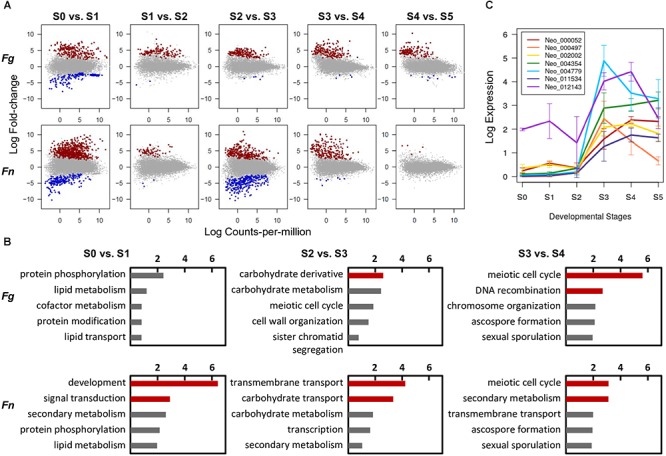
Conserved and divergent molecular events during perithecium development in *F. graminearum* (*Fg*) and *F. neocosmosporiellum* (*Fn*). **(A)** Differential expression (DE) analyses between two successive developmental stages [log_2_[fold-change] > 1.5, false discovery rate (FDR) 5%]. Note increase in DE genes in ‘S2 vs. S3’ comparison in *F. neocosmosporiellum.* **(B)** Top five represented GO terms in functional enrichment analyses for DE genes upregulated in later stage. Red bars indicate values of negative log_10_[adjusted *P*-value] for GO terms that are significantly enriched at 5% FDR. **(C)** Expression profiles of seven carbohydrate transporter genes differentially expressed and specifically induced in *Fn* at S3. Expression levels are presented as log_2_[RPKM+1].

To search for genes that play important roles in *F. neocosmosporiellum* and *F. graminearum*, we identified DE genes unique to each developmental stage. Overall, upregulated genes were predominant in advanced developmental stages in the differential expression analyses, suggesting that gene induction mechanisms represent a common means for gene regulation during perithecium development (Figure 4A). In *F. neocosmosporiellum*, perithecial volume dramatically increased within a relatively short period time between S2 and S3 (48 h). This process resulted in much larger perithecia when compared to *F. graminearum* (Figure 1C), which required 24 h for the same developmental interval. Accordingly, numerous DE genes were identified between S2 and S3 in *F. neocosmosporiellum*, indicating that many cellular processes were active (Figure 4A).

To identify the main cellular events that took place at each stage of perithecium development, we performed functional enrichment analyses for DE genes upregulated in advanced stages. As a result, genes from similar families were found to be enriched in both fusaria. For example, meiosis-related genes were commonly enriched for the GO term ‘meiotic cell cycle process’ at 5% FDR in both species when perithecia reached maturity and asci developed (Figure 4B). Nevertheless, we observed a striking difference in the ‘S2 vs. S3’ comparison, in which nutrient transport-related genes were significantly enriched for GO terms ‘transmembrane transport’ and ‘carbohydrate transport’ in *F. neocosmosporiellum*. The GO term ‘transmembrane transport’ had 30 child (direct descendent) GO terms with more specific annotations related to transport of various cellular substrates. Among them, we examined 15 GO terms relevant to fungi and found that *F. neocosmosporiellum* possessed approximately 50% more genes related to carbohydrate transmembrane transport (GO:0034219) in *F. graminearum* (67 vs. 44; Table 3). Orthologous gene cluster analyses showed that there were 26 carbohydrate transmembrane transport genes present in *F. neocosmosporiellum*, whereas most of the *F. graminearum* genes (40 out of 44) grouped with *F. neocosmosporiellum* orthologs. Among the 67 carbohydrate transporter genes in *F. neocosmosporiellum*, seven were DE and specifically induced at S3 (Figure 4C).

**Table 3:** Transporter genes expressed during perithecium development in *Fusarium graminearum* (*Fg*) and *F. neocosmosporiellum* (*Fn*).

Gene ontology ID number and description	*Fg*	*Fn*
GO:0055085 transmembrane transport	772*^a^* (839)*^b^*	917 (1196)
GO:0034219 carbohydrate transmembrane transport	44 (50)	67 (87)
GO:1901264 carbohydrate derivative transport	83 (91)	95 (128)
GO:0071806 protein transmembrane transport	44 (44)	48 (51)
GO:1901679 nucleotide transmembrane transport	55 (61)	61 (85)
GO:0015780 nucleotide-sugar transmembrane transport	5 (5)	5 (5)
GO:0006855 drug transmembrane transport	28 (28)	34 (39)
GO:0035382 sterol transmembrane transport	1 (1)	1 (1)
GO:0035461 vitamin transmembrane transport	8 (10)	7 (17)
GO:1903825 organic acid transmembrane transport	129 (142)	157 (216)
GO:0044718 siderophore transmembrane transport	2 (2)	3 (3)
GO:0034220 ion transmembrane transport	314 (336)	368 (454)
GO:0034486 vacuolar transmembrane transport	15 (15)	15 (17)
GO:1990542 mitochondrial transmembrane transport	47 (47)	49 (51)
GO:0098739 import across plasma membrane	18 (22)	23 (28)
GO:0034762 regulation of transmembrane transport	48 (52)	49 (55)

^a^*Filtered by an expression threshold (see Materials and Methods).*^b^*Total predicted genes in parentheses (*E*-value < 10^*-5*^).*

### Secondary Metabolite Biosynthetic Genes

Functional enrichment analyses indicated that many secondary metabolism-related genes were upregulated during perithecium development in *F. neocosmosporiellum*, especially during later stages (S3 vs. S4; Figure 4B). Using antiSMASH, we identified a total of eleven PKSs, nine NRPSs, and three terpene biosynthesis-related genes (TCs) in the *F. neocosmosporiellum* genome. Among these, a total of eight PKSs, four NRPSs, and three TCs genes were expressed in our transcriptome datasets (Table 4). Orthologs of all of the expressed secondary metabolite-related genes, except an *NRPS* gene (*SimA*), occur in the closely related species *F. euwallaceae* and *F. solani* f. sp. *pisi*, which are members of the FSSC. The proteins encoded by these genes shared amino acid (AA) sequence similarities ranging from 80 to 98% (Table 4). An *NRPS* gene homologous to *SimA* was predicted to encode a 15,274 AA-long polypeptide, showing 96% sequence similarity with the cyclosporine synthetase SimA in *Tolypocladium inflatum* (Bushley et al., 2013).

**Table 4:** Secondary metabolite biosynthetic genes.

*F. neocosmosporiellum^a^*	Best hit in *F. graminearum^a^*	%*^b^*	Best hit in NCBI database*^c^*	%*^b^*
PKS2 (Neo_003331)	PKS2 (FGRRES_04694)	55	*F. euwallaceae* (ALQ32835)	82
PKS3 (Neo_007042)	PKS3 (FGRRES_17168)	76	*F. euwallaceae* (ALQ32837)	95
PKS7 (Neo_006055)	PKS7 (FGRRES_08795)	62	*F. euwallaceae* (ALQ32840)	80
PKS22 (Neo_009895)	PKS11 (FGRRES_01790)	42	*F. euwallaceae* (ALQ32841)	93
PKS30 (Neo_001289)	PKS5 (FGRRES_17677)	36	*F. euwallaceae* (ALQ32842)	90
PKS31 (Neo_001313)	PKS11 (FGRRES_01790)	41	*F. solani* f.sp. *pisi* (XP_003041774)	87
PKS33 (Neo_000291)	PKS11 (FGRRES_01790)	52	*F. euwallaceae* (ALQ32839)	95
PKS35 (Neo_002749)	PKS12 (FGRRES_02324)	44	*F. solani* f.sp. *pisi* (AAS48892)	93
NRPS2 (Neo_002267)	NRPS2 (FGRRES_05372)	65	*F. solani* f.sp. *pisi* (XP_003044019)	91
NRPS6 (Neo_004434)	NRPS6 (FGRRES_03747)	78	*F. solani* f.sp. *pisi* (XP_003043406)	96
NRPS27 (Neo_005330)	NRPS6 (FGRRES_03747)	45	*F. solani* f.sp. *pisi* (XP_003048492)	94
SimA (Neo_003447)	NRPS19 (FGRRES_15676)	43	*Tolypocladium inflatum* (CAA82227)	96
TC1 (Neo_002462)	ERG9 (FGRRES_09381)	87	*F. solani* f.sp. *pisi* (XP_003053888)	98
TC2 (Neo_003698)	TRI5 (FGRRES_03537)	42	*F. solani* f.sp. *pisi* (XP_003047531)	90
TC3 (Neo_003381)	– (FGRRES_16578)	81	*F. solani* f.sp. *pisi* (XP_003050574)	96

^a^*Annotations of *PKS* and *NRPS* genes, respectively, as in Hansen et al. (2015) and Brown and Proctor (2016).*^b^*Percent similarity of deduced polypeptides sequence. *^*c*^*BLASTp search results performed on November 16, 2018. Accessions are in parenthesis.*

**Figure 5:**
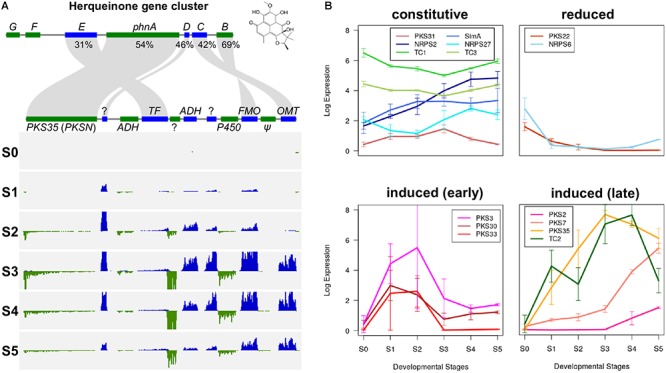
Secondary metabolism in *F. neocosmosporiellum*. **(A)** Herqueinone gene cluster consists of seven genes (*phnA*—*phnG*) in *Penicillium herquei* (top) compared with *PKS35* (aka. *PKSN*) gene cluster in *F. neocosmosporiellum* (bottom). Percent amino acid sequence similarity of conserved genes is presented. Per-base coverage for RNA-seq reads is shown for both DNA strands (blue and green) during perithecium development (S0—S5). *ADH*, alcohol dehydrogenase; *FMO*, Flavin-dependent monooxygenase; *OMT*, *O*-methyltransferase; *PKS*, polyketide synthase; *TF*, transcription factor; ?: hypothetical protein (no predicted protein domain found in BLASTp search), *Ψ*: likely pseudogene (no expression). **(B)** Gene expression profiles of secondary metabolite biosynthetic genes during perithecium development. Four non-ribosomal peptide synthetases (NRPSs), 8 polyketide synthases (PKSs) and 3 terpene biosynthesis-related genes (TCs) are grouped according to their expression patterns, and presented as log_2_[RPKM+1].

Some PKS and NRPS genes in *F. neocosmosporiellum* and *F. graminearum* were orthologous, while others were not (Table 4). In *F. graminearum*, *PKS3* (also known as *PGL1* and *FSR1*) has been shown to be involved in synthesis of the black pigment in the perithecial walls (Gaffoor et al., 2005; Frandsen et al., 2016). However, sexually reproducing species in the FSSC, including *F. neocosmosporiellum*, produce orange to red perithecia. *PKSN* was reported to be responsible for the red pigment in the perithecial walls in another phylospecies within the FSSC (Graziani et al., 2004). AntiSMASH identified a *PKSN* homolog in *F. neocosmosporiellum* (henceforth *PKS35*; Brown and Proctor, 2016) and predicted the *PKS35* gene cluster. The boundaries of this putative cluster were further supported by the co-expression of genes within it (Figure 5A). We identified ten co-regulated genes, including the *PKS35*, whose expression peaked at ‘S3’, when pigmented perithecial walls form (Figure 5A). Interestingly, five of the genes in the putative *PKS35* gene cluster are homologs of the five genes in the gene cluster responsible for biosynthesis of herqueinone, a red pigment produced by *Penicillium herquei* (Stodola et al., 1951; Gao et al., 2016) (Figure 5A).

In *F. neocosmosporiellum*, secondary metabolite biosynthetic (SM) genes that were expressed in our transcriptome data could be categorized by their expression patterns: (1) constitutive expression during all stages of perithecium development, (2) reduced after sexual induction, (3) induced at earlier stages (S1–S2), and (4) induced at later stages (S3–S5) (Figure 5B). Several SM genes, including the *SimA*, exhibited constitutive expression at all stages of perithecium development. *PKS3*, *PKS30*, and *PKS33* were induced at earlier developmental stages and declined afterward. By comparison, expression of *PKS2*, *PKS7*, *PKS35*, and *TC2* peaked at later developmental stages.

**Figure 6:**
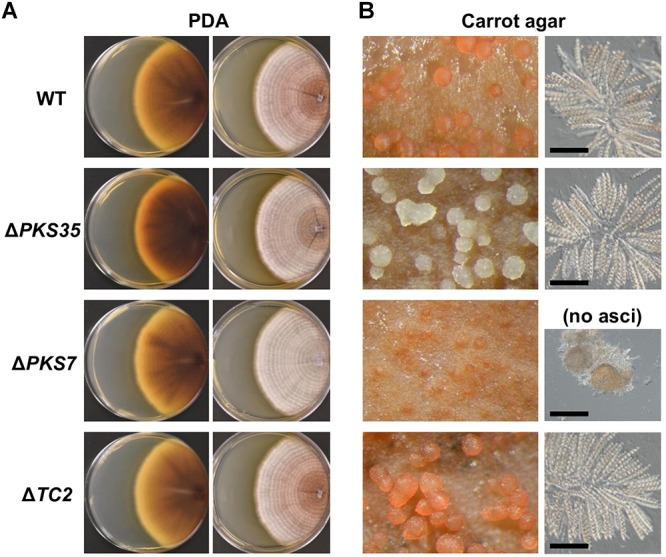
Knockout phenotypes of secondary metabolite biosynthesis genes induced at later stages of perithecium development. **(A)** Colony morphologies of deletion mutants (Δ*PKS35*, Δ*PKS7*, and Δ*TC2*) grown on potato dextrose agar [views of culture from above (left); from below (right)]. **(B)** Perithecium production on carrot agar (left). Asci released from a squashed perithecium (right). Note that Δ*PKS7* produced protoperithecia but mature asci failed to develop. Bar = 100 μm.

To determine whether SM genes have a role in perithecium development, we selected three *PKS7*, *PKS35*, and *TC2* for functional analysis by targeted gene deletion, which were highly induced at the later stages of development (Figure 5B). The metabolic products of *PKS7*- and *TC2*-encoded enzymes are not known, whereas the *PKS35*-encoded enzyme is required for production of a red perithecial pigment (Graziani et al., 2004). Deletion of *PKS35* did not cause any visible change in growth, asexual sporulation, or ascospore formation. However, perithecia produced by the Δ*PKS35* were albino, suggesting a specific role of *PKS35* in perithecial wall pigmentation in *F. neocosmosporiellum* (Figure 6). Deletion of *PKS7* caused aborted perithecium development, leading to production of smaller perithecia that were devoid of asci (Figure 6). This defective phenotype was restored by genetic complementation of the Δ*PKS7* with the wild-type copy of *PKS7* (Supplementary Figure S1). Deletion of *TC2* did not cause any detectable morphological differences (Figure 6).

## Discussion

We used a comparative genomic and transcriptomic approach to functionally analyze an organism whose life history remains unclear. Our study centered on the perithecium development, because fruiting bodies provide the primary long distance dispersal and long-term survival propagules, especially for ascomycetes fungi, and because *F. neocosmosporiellum* produces prolific perithecia in culture. Formation of sexual fruiting bodies is a crucial step in the life cycle of many fungi. It requires sensing of environmental cues to initiate and complete development fruiting bodies and to disperse sexual spores produced within them. Fruiting body form and function is diverse, even among species in the genus *Fusarium*, and the diverse mechanisms have evolved that facilitate ascospore dispersal and survival. Perithecia of *F. neocosmosporiellum* and *F. graminearum* have distinct features, which may have ecological relevance. These features include: (1) production of round, single-celled ascospores, instead of ellipsoidal, multiseptate ascospores of many *Fusarium* spp. (Smith, 2007); (2) the majority of spores are released through cirrhi that are most likely splash-dispersed; and (3) perithecia are larger than those produced by *F. graminearum* (300 μm in *F. neocosmosporiellum* compared with 180 μm in *F. graminearum*) (Calaça et al., 2013; Gabriel et al., 2013; Sun et al., 2014).

Ellipsoidal ascospores that are forcibly discharged are prevalent throughout the Ascomycota and in most perithecium-producing *Fusarium* species (Zhou et al., 2018). This shape is optimized for low drag (Roper et al., 2008). However, those produced by *F. neocosmosporiellum* are globose-to-subglobose, which may be explained by the loss of the ability to produce septate ascospores (O’Donnell, 2000). We have shown that the majority of the ascospores produced by *F. neocosmosporiellum* are not forcibly discharged; instead they accumulate in a cirrhus. From the pattern of deposition of spores forcibly discharged, we can infer that ascospores remain attached after they are discharged, and that they are likely held together by a mucilaginous droplet during flight. Production of a large, multi-ascospore projectile could overcome air drag that would be effective on a single spore, due to a larger projectile force from the increased mass. In *F. graminearum*, by contrast, ascospores disperse from each other after they are forcibly discharged from perithecia. In addition, ascospores discharged from *F. graminearum* perithecia are not coated in mucilage, as they are in *F. neocosmosporiellum*. As a result, when dispersed onto a glass surface, *F. graminearum* ascospores are easily washed off (Trail et al., 2005), whereas *F. neocosmosporiellum* are not.

We have shown that *F. graminearum* spores are launched an average of 0.6 cm in the chamber we used in this study (Trail et al., 2005). A group of reports summarized by Calaça et al. (2013) indicate *F. neocosmosporiellum* is a coprophilous fungus based on its recovery from a variety of herbivore dung in Africa, Italy and Brazil. *Coprophilous ascomycetes* fungi often share several characteristics (Krug et al., 2004). They tend to discharge large projectiles that are capable of escaping the “zone of repugnance” where some spores by chance land on vegetation and may be ingested by herbivore. The ascospores of *F. neocosmosporiellum* have unusually thick walls (Calaça et al., 2013), which may serve as protection against the harsh environment of a herbivore gut. In addition, the presence of larger numbers of lysozyme genes (GH24 and GH25 families) that we found in the genome of *F. neocosmosporiellum* might enhance its competitiveness on dung.

The level of *MAT* gene expression appears to be correlated with fertility in *F. graminearum* (Kim et al., 2012). Interestingly, three strains of the closely related species, *F. asiaticum*, which is also a member of the FSAMSC, exhibited lower fertility, and their *MAT* gene expression levels were significantly lower than *F. graminearum*t (Kim et al., 2012). *Fusarium neocosmosporiellum* NRRL 22166 is highly fertile. All five *MAT* genes are highly induced in a coordinated manner, producing abundant perithecia on diverse agar media, including PDA, on which *F. graminearum* does not produce perithecia. The observed difference in *MAT* gene expression patterns between the two fusaria suggests different regulatory networks, including those for *MAT* genes during perithecium development. The plant pathogen *F. graminearum* discharges ascospores when the temperature is warmer, which coincides when hosts are available (Guenther and Trail, 2005). Additionally, *F. graminearum* can produce perithecia in association with specific host cells (Guenther and Trail, 2005). The timing and location of perithecium production by *F. neocosmosporiellum* in nature are not known; however, our findings suggest that they may accommodate variable conditions.

CAZymes are essential components of fungal cells because they facilitate absorptive nutrition. Coordination of a set of CAZymes is required to liberate monomeric sugars from plant biomass, along with accessory enzymes that cleave side chains of poly- and oligosaccharides. We found that sets of co-expressed CAZyme genes were induced in different stages of perithecium development, indicating that they appear to act cooperatively and play stage-specific developmental roles in *F. graminearum* and *F. neocosmosporiellum*. The type of CAZymes encoded in fungal genomes and their expression at different stages of the life cycle are potential clues to the nutrient sources they require. Recent comparative genomic analyzes revealed that carbon utilization is not correlated with CAZyme content in the aspergilli (Vesth et al., 2018). The authors suggested that differences in their ability to degrade plant biomass is reflected by the level of CAZymes gene expression rather than copy number. Although CAZyme content was largely conserved between *F. graminearum* and *F. neocosmosporiellum*, some CAZyme families showed different copy numbers and divergent expression patterns in the latter species. We discovered several CAZyme families in *F. neocosmosporiellum* that offered clues to its ecology. These included CAZymes that targeted plant cell wall components, such as cellulose and pectin, and lysozymes targeting bacterial cells. Collectively, these enzymes suggest that *F. neocosmosporiellum* is well adapted to life as a saprotroph, soil-dwelling, coprophile, and weak phytopathogen.

The plant pathogen *F. graminearum* colonizes host plants by penetrating intercellular spaces rich in pectin (Jansen et al., 2005) and produces perithecia on host tissues after the host has senesced (Trail, 2009). The three putative pectin acetylesterases (CAZyme family CE12) involved in pectin degradation were highly induced during the early developmental stage on carrot agar medium, suggesting that *F. graminearum* may be more efficient at using pectin as a carbon source in this earlier stage. By way of comparison, *F. neocosmosporiellum* appears to have lost some of the CAZyme families related to degradation of plant biomass, such as hemicellulose and cutin (CAZyme family CE5). Loss of cutinases may indicate a change in the mode of pathogenicity, although the role for cutinase in virulence of *F. solani* f. sp. *pisi* on garden pea has been controversial (Stahl and Schafer, 1992; Rogers et al., 1994; Stahl et al., 1994). However, in *Magnaporthe grisea* mutation of the Cutinase2 gene (*CUT2*) attenuated virulence (Skamnioti and Gurr, 2007, 2008). *F. neocosmosporiellum* possesses three cellulases (CAZyme family GH5), compared with only one in *F. graminearum*, and two of them are induced at early or late developmental stages. In addition, genes for three lysozymes were found in *F. neocosmosporiellum*, targeting bacterial cell walls (i.e., peptidoglycan), which are absent in *F. graminearum*. Among them, Neo_008641 (GH25) has not been reported in other *Fusarium* species. These enzymes might facilitate survival in the ecological niches that *F. neocosmosporiellum* occupies, supporting a predominantly saprotrophic lifestyle.

Divergence of the transport-related gene repertoire has contributed to evolution of fungal fruiting bodies (Nguyen et al., 2017). *F. neocosmosporiellum* NRRL 22166 possesses more nutrient transport-related genes than *F. graminearum* PH-1, and the transcriptome data shows that many of these genes in the former species are specifically induced between S2 and S3. This observation suggested that *F. neocosmosporiellum* may require active transport of carbon and other nutrients, which may account for the large increase in perithecium volume in a relatively short time period. Interestingly, a *F. graminearum* knockout mutant lacking a MYB-family transcription factor gene produces larger perithecia that were approximately the same size as those of *F. neocosmosporiellum* (Lin et al., 2012). In eukaryotes, MYB-family proteins are involved in cell proliferation and differentiation (Oh and Reddy, 1999). In our transcriptome data, expression of the *MYT2* ortholog in *F. neocosmosporiellum* was high at S1, decreased at S2, but was high again at S3. However, expression of *MYT2* in *F. graminearum* remained highfrom S1 through S3 (data not shown). These expression patterns suggest that *MYT2* might function as a negative regulator of cell proliferation during perithecium development, presumably by controlling nutrient transport-related genes. Furthermore, the decreased expression level of the *MYT2* ortholog at S2 may explain why *F. neocosmosporiellum* produces perithecia that are larger than in *F. graminearum*. To test this hypothesis, we attempted to generate *MYT2*–deletion mutants in *F. neocosmosporiellum*. However, several unsuccessful attempts to obtain the mutant suggests that the *MYT2* ortholog in *F. neocosmosporiellum* is essential.

Secondary metabolites are often implicated in niche adaptation and host specialization especially for pathogens or parasites (Turgeon and Bushley, 2010). Various fungi within the Hypocreales, including *F. neocosmosporiellum*, have been reported to produce cyclosporines, which are used as immunosuppressant drugs (Borel et al., 1977). Here, we identified a full-length cyclosporine synthetase gene, *SimA* (45,825 bp-long), for the first time in the Nectriaceae. Cyclosporines are produced by many insect parasites, such as *Beauveria* spp. and *Tolypocladium inflatum*, and are important virulence factors against susceptible insect hosts (Yang et al., 2018). Though presently unknown, frequent isolation of *F. neocosmosporiellum* from soybean cyst nematodes may be due to the toxic properties of cyclosporines (Gintis et al., 1983).

Orthologs of *F. neocosmosporiellum* PKS genes are present in other members of the FSSC, including *F. solani* f. sp. *pisi* and *F. euwallaceae* (80–95% AA sequence similarity). Of these, *PKS7* exhibited the least sequence identity (80%) among FSSC members and appears to be fast-evolving. Despite the sequence divergence, the protein sequence and domain structure of *PKS7* in *F. neocosmosporiellum* is conserved with the orthologous gene in *F. graminearum*. Interestingly, a deletion mutant of *PKS7* in *F. graminearum* also failed to develop mature perithecia, suggesting a conserved function of the yet-unknown PKS products in perithecium development (Kim H.-K. et al., 2015). We are currently trying to determine the metabolic product(s) of the *PKS7* gene cluster and its role in perithecium development.

The results of our study demonstrate that *PKS35* is required for the red pigmentation of *F. neocosmosporiellum*, as has been reported previously for another member of the FSSC (Graziani et al., 2004). Our analysis also revealed that the *PKS35* cluster includes a flavin-dependent monooxygenase gene with 69% deduced AA sequence identity to PhnB, the enzyme catalyzes the final step in biosynthesis of the red pigment herquinone. This final step is essential for formation of the three-ring phenalenone structure that makes up a large part of the herquinone molecule. In addition, the domain structure of PKS35 and the herquinone PKS, PhnA, are nearly identical. These observations suggest that the two red pigments, one that accumulates in the perithecial wall of *F. neocosmosporiellum* and the other secreted by hyphae of *Penicillium herquei*, might share a common evolutionary origin and core chemical structure. In addition, *PKS35* orthologs have been reported in several members of the FSSC (Hansen et al., 2015; Brown and Proctor, 2016). Although red pigments produced by land plants are thought to attract insects (Tanaka et al., 2008), future studies are needed to determine whether those produced in the perithecia of *F. neocosmosporiellum* have a similar function.

Throughout the last century, much emphasis has been placed on the study of life histories of disease-causing plant pathogens. More recently, scientists have combined molecular genetics and genomics tools to identify specific genes involved in pathogenicity. Here, we have used a comparative genomic and transcriptomic approach to provide a functional view of perithecium development and to identify biological clues into the life history of *F. neocosmosporiellum*, a cosmopolitan fungus that has been found in a variety of niches, including dung and soil. Furthermore, *F. neocosmosporiellum* ascospores have morphological characteristics that are similar to other dung fungi. In summary, this study revealed transcriptional programming that accompanied production of large perithecia and secondary metabolites linked to perithecium development, processes that may facilitate survival and dispersal of this cosmopolitan fungus in nature.

## Author Contributions

WK and FT conceived and designed the experiments. WK and FT performed the experiments. KO and RP sequenced the genome. BC annotated the genome. WK and FT analyzed the data and wrote the manuscript. JT, KO, and RP edited the manuscript. FT and JT conceived of the larger project which includes this study, and obtained the funding.

## Conflict of Interest Statement

The authors declare that the research was conducted in the absence of any commercial or financial relationships that could be construed as a potential conflict of interest.

## Footnotes

1 https://rtsf.natsci.msu.edu/genomics

2 www.bioinformatics.babraham.ac.uk/projects/fastqc
