# Supplementary figures and images for: Comparative Genomics and Transcriptomics During Sexual Development Gives Insight Into the Life History of the Cosmopolitan Fungus Fusarium neocosmosporiellum
Source: Front Microbiol. 2019 Jun 7;10:1247. doi: 10.3389/fmicb.2019.01247 (PMC6568001; doi:10.3389/fmicb.2019.01247)

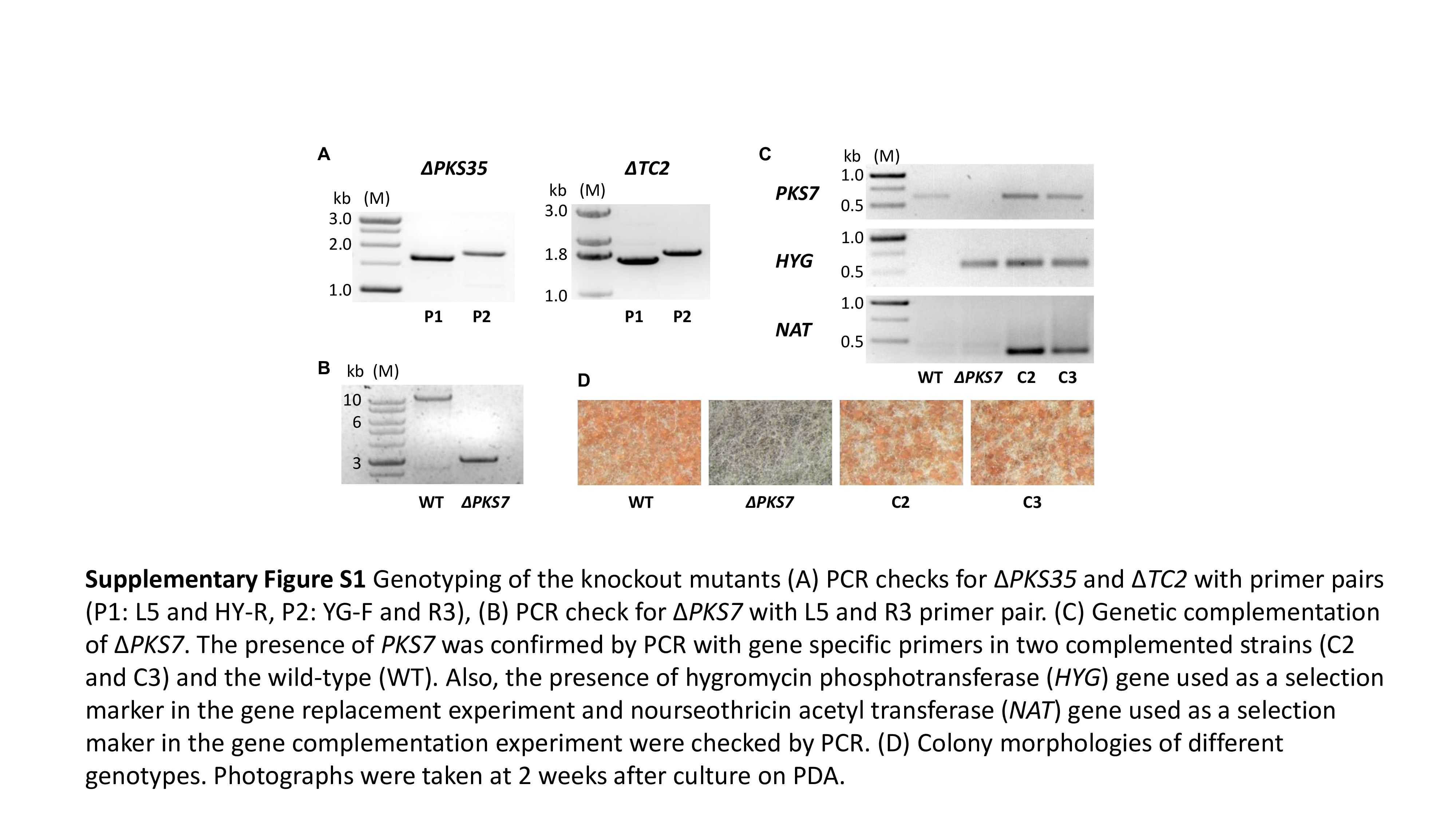

Supplement: Supplementary file 3 [file Image_1.JPEG]

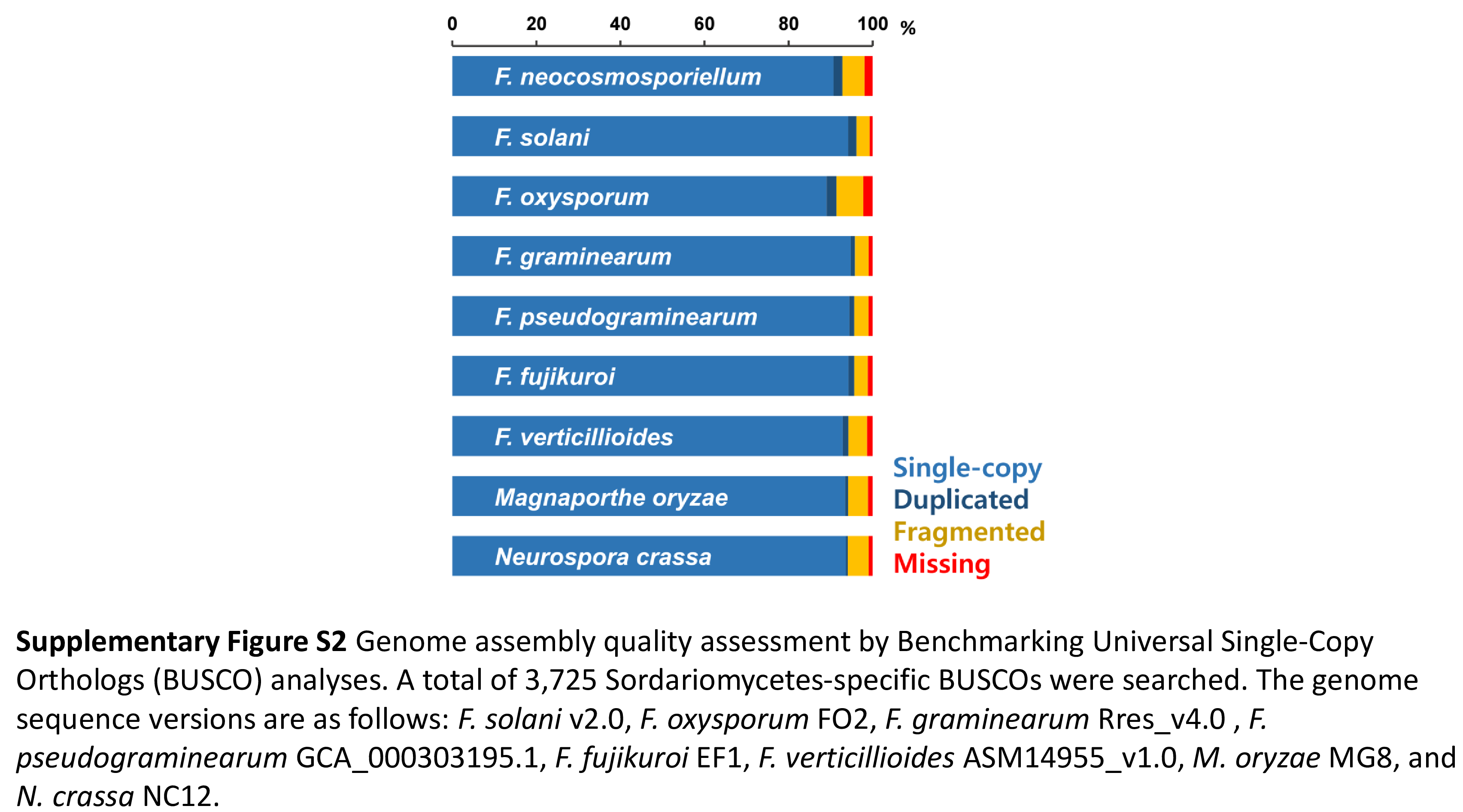

Supplement: Supplementary file 4 [file Image_2.JPEG]

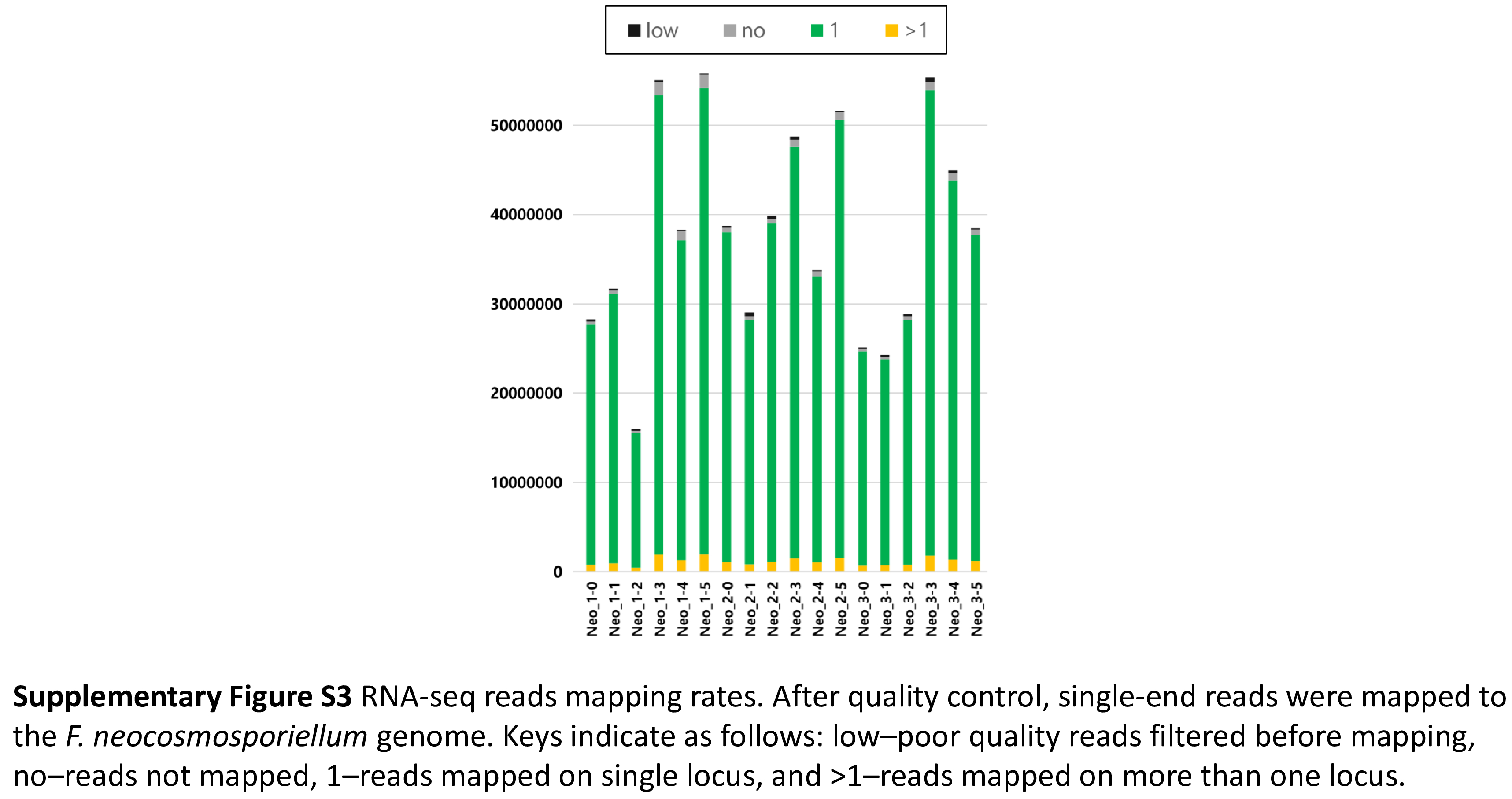

Supplement: Supplementary file 5 [file Image_3.JPEG]
